# Multi-centred mixed-methods PEPFAR HIV care & support public health evaluation: study protocol

**DOI:** 10.1186/1471-2458-10-584

**Published:** 2010-09-29

**Authors:** Richard Harding, Victoria Simms, Suzanne Penfold, Paul McCrone, Scott Moreland, Julia Downing, Richard A Powell, Faith Mwangi-Powell, Eve Namisango, Peter Fayers, Siân Curtis, Irene J Higginson

**Affiliations:** 1 King's College London, Cicely Saunders Institute Department of Palliative Care, Policy and Rehabilitation School of Medicine at Guy's, King's and St Thomas' Hospitals Bessemer Road, London SE5 9PJ, UK; 2 King's College London Department of Health Service and Population Research Institute of Psychiatry Box P024, De Crespigny Park London, SE5 8AF, UK; 3 Futures Group One Thomas Circle, NW, Suite 200 Washington DC 20005, USA; 4 African Palliative Care Association PO Box 72518 Kampala, Uganda; 5 University of Aberdeen Department of Public Health, School of Medicine Polwarth Building Foresterhill, Aberdeen AB25 2ZD, UK; 6 MEASURE Evaluation Project Carolina Population Center University of North Carolina at Chapel Hill, CB 8120 Chapel Hill, NC 27599, USA

## Abstract

**Background:**

A public health response is essential to meet the multidimensional needs of patients and families affected by HIV disease in sub-Saharan Africa. In order to appraise curret provision of HIV care and support in East Africa, and to provide evidence-based direction to future care programming, and Public Health Evaluation was commissioned by the PEPFAR programme of the US Government.

**Methods/Design:**

This paper described the 2-Phase international mixed methods study protocol utilising longitudinal outcome measurement, surveys, patient and family qualitative interviews and focus groups, staff qualitative interviews, health economics and document analysis.

Aim 1) To describe the nature and scope of HIV care and support in two African countries, including the types of facilities available, clients seen, and availability of specific components of care [Study Phase 1]. Aim 2) To determine patient health outcomes over time and principle cost drivers [Study Phase 2].

The study objectives are as follows. 1) To undertake a cross-sectional survey of service configuration and activity by sampling 10% of the facilities being funded by PEPFAR to provide HIV care and support in Kenya and Uganda (Phase 1) in order to describe care currently provided, including pharmacy drug reviews to determine availability and supply of essential drugs in HIV management. 2) To conduct patient focus group discussions at each of these (Phase 1) to determine care received. 3) To undertake a longitudinal prospective study of 1200 patients who are newly diagnosed with HIV or patients with HIV who present with a new problem attending PEPFAR care and support services. Data collection includes self-reported quality of life, core palliative outcomes and components of care received (Phase 2). 4) To conduct qualitative interviews with staff, patients and carers in order to explore and understand service issues and care provision in more depth (Phase 2). 5) To undertake document analysis to appraise the clinical care procedures at each facility (Phase 2). 6) To determine principle cost drivers including staff, overhead and laboratory costs (Phase 2).

**Discussion:**

This novel mixed methods protocol will permit transparent presentation of subsequent dataset results publication, and offers a substantive model of protocol design to measure and integrate key activities and outcomes that underpin a public health approach to disease management in a low-income setting.

## Background

Within Sub-Saharan Africa during 2007 There were an estimated 22 million individuals living with HIV infection, 1.5 million HIV-related deaths, and 11.6 million children orphaned due to parental HIV infection [1]. A public health approach is clearly required to provide appropriate health care to meet the preventive, care and support, treatment, and bereavement needs associated with the epidemic. In terms of domains of interest for a public health approach, the endpoints must include both the patient and their informal carers and family. This is particularly true in resource limited settings where progressive disease compounds family-wide poverty, and where a limited health sector relies on informal caregivers to provide both inpatient and home care. Further, we must determine the effectiveness of interventions across the multiple dimensions of need that underpin the lived experience of disease, i.e. the physical (e.g. virological responses, symptoms and treatment side effects), psychological (e.g. anxiety and depression), social (family needs, food security and stigma) and spiritual needs (e.g. finding peace and comfort through spiritual care). These domains are inextricably linked, and arguably cannot be effectively addressed in isolation.

In 2003 the United States government (USG) funded a five-year, $15 billion initiative to combat the global HIV/AIDS epidemic: the President's Emergency Plan for AIDS Relief (PEPFAR). The funds were allocated approximately as follows: treatment (55%), prevention (20%), assisting orphans and vulnerable children (10%) and care and support of individuals with HIV/AIDS (15%) for years 2003-2008. In 2008, PEPFAR was reauthorized for a further five years up to $48 billion.

Evaluation of the effect of PEPFAR funding in its target countries has established that there has been a decrease in HIV-related deaths [2], and a reduction in the number of HIV positive births [3]. While the focus on increased access to treatment has achieved results, there has been a lack of evidence for the effectiveness of care and support on patient and family self-report outcomes such as quality of life, or on evaluation of the models of care being delivered. Lack of attention to these outcomes may undermine and diminish the gains brought by improved treatment access. Person-centred self report measures are essential as there is an increasing body of evidence that people living with HIV infection endure multiple distressing problems from the point of diagnosis and alongside treatment [4], including suicidal ideation [5], depression [6,7], fatigue [8], poverty, malnutrition [9], pain [10] and other symptoms [11], poor access to symptom controlling drugs [12], spiritual distress [13] and information needs [14].

In order to evaluate funded programmes, and to facilitate evidence-based programming of the funding response, Public Health Evaluations (PHEs) have been commissioned in line with allocation of funding detailed above. The Care and Support PHE reported here has developed a multi-centred, two phase mixed-methods international study protocol. We report the protocol to provide access to the full study methods [15], and to offer examples of our responses to the methodological challenges of conducting longitudinal multi-methods and multidimensional research.

## Methods/Design

### Aims and objectives

This PHE has two aims. These are as follows. Aim 1) To describe the nature and scope of HIV care and support in two African countries, including the types of facilities available, clients seen, and availability of specific components of care [Study Phase 1]. Aim 2) To determine patient health outcomes over time and principle cost drivers [Study Phase 2].

The study objectives are as follows. 1) To undertake a cross-sectional survey of service configuration and activity by sampling 10% of the facilities being funded by PEPFAR to provide HIV care and support in Kenya and Uganda (Phase 1) in order to describe care currently provided. 2) To conduct patient focus group discussions at each of these (Phase 1) to determine care received. 3) To undertake a longitudinal prospective study of 1200 patients who are newly diagnosed with HIV or patients with HIV who present with a new problem attending PEPFAR care and support services. Data collection includes self-reported quality of life, core palliative outcomes and components of care received (Phase 2). 4) To conduct qualitative interviews with staff, patients and carers in order to explore and understand service issues and care provision in more depth (Phase 2). 5) To undertake document analysis to appraise the clinical care procedures at each facility (Phase 2). 6) To determine principle cost drivers including staff, overhead and laboratory costs (Phase 2).

### Methods/Design

The study has a mixed methods design across two Phases.

The first Phase utilises survey methods, focus group discussions, document analysis and pharmacy checklist review among a randomly selected stratified sample of all HIV facilities receiving PEPFAR funds in Kenya and Uganda.

The second Phase at each site employs qualitative interviews with patients, family caregivers and staff, longitudinally applied outcome tools among patients in each of 12 facilities across Kenya and Uganda, and a costing study.

**Figure 1:**
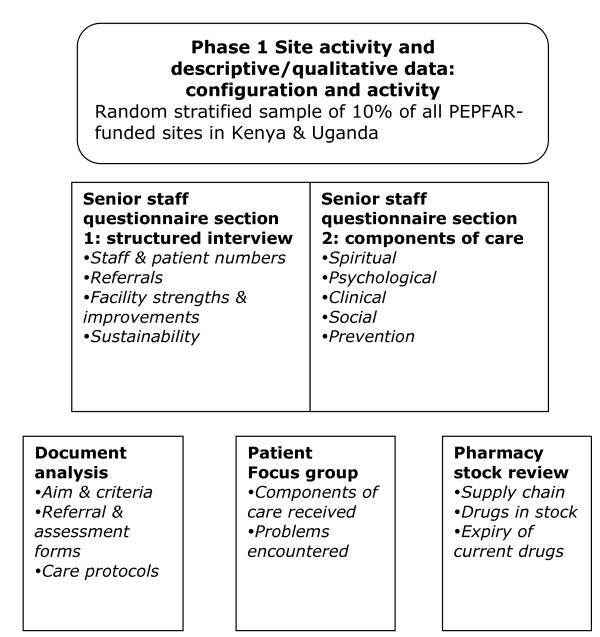
**Study flow chart for Phase 1: Structured descriptive analysis of facility configuration and activity**.

**Figure 2:**
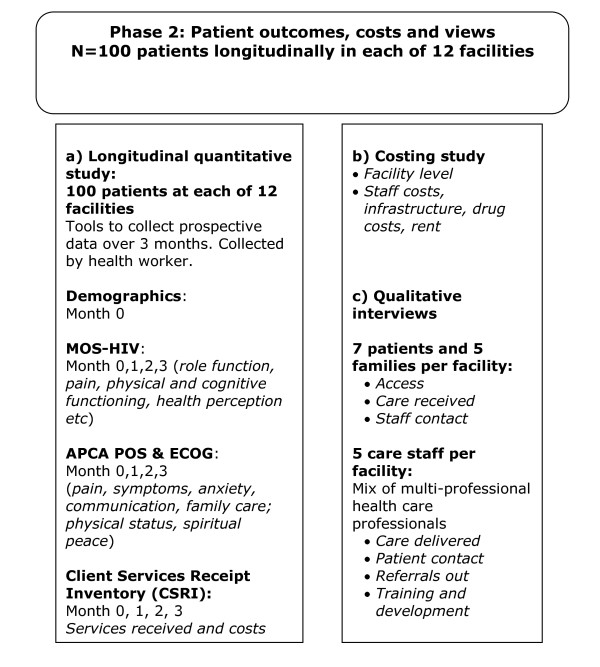
**Study flow chart for Phase 2: Longitudinal outcome evaluation**.

The study protocol is depicted in Figures 1 and 2

### Protocol development

Kenya and Uganda are two of the 15 PEPFAR 'focus countries'. They are both low-income countries with a high prevalence of HIV, and are countries with public health systems that may offer lessons for replication in other African countries.

The PEPFAR programme is country-wide in the two countries engaged in this PHE (and had been implemented for several years before the evaluation was commissioned. Therefore it was not possible to conduct a 'before and after' study. One potential study design option was to compare outcomes at facilities receiving PEPFAR funding with those which did not, but there are few large facilities in the target countries which have never received PEPFAR funding, and they would have little motivation to participate. Little information exists regarding the quality of life of general populations, which could have been used as a comparison sample. Further, facilities did not have stated targets against which their performance could be compared.

A cross-sectional study would only be able to identify differences between facilities, which might be caused by population factors as well as variations in care and support delivery.

Therefore, a longitudinal cohort study design has been selected to allow the effect of care over time to be examined. This design offers the most feasible and robust option for evaluation of outcomes, although it is not possible to remove the effects of previous contact with PEPFAR Care and Support.

Patient self-reported health has been selected as the outcome of interest because care and support aims to improve quality of life, and could not be properly assessed without measuring this outcome. A mixed-methods design incorporating both quantitative and qualitative methods allows triangulation and greater understanding of the data and its context.

This evaluation of PEPFAR-funded care and support for HIV is led by King's College London (KCL, Principal Investigator), in collaboration with MEASURE Evaluation at the University of North Carolina (UNC), the African Palliative Care Association (APCA) and the Kenyan Hospice and Palliative Care Association (KEHPCA). The aims, methods and implementation of the evaluation have been planned and agreed in consultation with the members of United States Government (USG) Care and Support Technical Working Group, Kenyan and Ugandan PEPFAR Country Teams, and representatives of the Ministries of Health in Kenya and Uganda.

### Ethical approval and procedures

Ethical approval to undertake the study has been received from the Ugandan National Council for Science and Technology (UNCST, Ref SS 1964), the Kenyan Medical Research Institute (Ref KEMRI/RES/7/3/1) and the College Research Ethics Committee at KCL (Ref CREC/06/07-140). Subsequent tool changes following initial piloting have also been approved.

During longitudinal data collection, all questionnaires are stored separately from consent forms, in a locked filing cabinet at the facility. Upon completion of the study, anonymised questionnaires are taken from the facility to the APCA offices for storage in locked filing cabinets. These arrangements are in line with ethical guidance and the UK Data Protection Act 1998. Interview transcripts are stored without any identifying information and names etc are deleted from the transcript. Original digital recordings are deleted once transcribed. All study computers are password protected.

#### Phase 1 methods

### Sampling

Of around 600 PEPFAR-funded HIV care facilities in each country, 60 have been selected for inclusion in the study (approximately 10% of PEPFAR-funded facilities). The inclusion criterion for eligibility in Phase 1 are that they receive PEPFAR funding to provide HIV care and support. The exclusion criteria are facilities that are paediatric-only or inaccessible (e.g. insecure, no road access). Facilities which do not meet the criteria above were replaced using the same random process (and replacements reported against the criteria).

According to routine monitoring patient numbers, the PEPFAR-funded care and support facilities include many smaller facilities. In order to capture a range of facility sizes within the study population, facilities were stratified by number of patients seen for HIV care in the previous financial year (according to national PEPFAR records) and divided into three strata (1 to 100, 101 to 500 and >500 patients seen), resulting in unequal and calculable sampling fractions. Twenty facilities were randomly sampled within each of the strata for the study population.

### Tool development & data collection procedure

All tools were developed by a multidisciplinary team, including medical professionals, expert HIV researchers and programmers, in conjunction with USG Care and Support Technical Working Group and the PEPFAR country teams. All tools have been piloted in one large and one small Phase 1 facility in Uganda. These facilities were two of the 60 selected, and data from the pilot utilised in the final analyses. Following piloting, the wording and structure of the tools were modified and clarified.

Facilities are informed of the planned survey through the Ministry of Health (MOH) and invited to participate. Local African researchers attend each sampled site to collect data on a pre-arranged day. Data are recorded on two separate sets of identical forms. One set is left with the facility while the other is taken by the researchers for data entry.

Four data collection tools are used.

#### Phase 1 data collection tool 1: Senior staff interview

The local researchers interview a group of senior staff, including facility managers and senior clinical staff, at each health facility to collect responses to closed and open-ended questions about patient numbers, infrastructure and staffing. This tool also includes a version of the Client Services Receipt Inventory (CSRI) (Beecham and Knapp 2001) adapted for the aims of this study and the HIV setting in Africa to identify services offered to patients with HIV. The CSRI asks if the facility offers various specific components of care under the four domains of care: clinical, psychological, spiritual, social and preventive. The tool is designed for use across the wide range of size and type of HIV care facilities funded by PEPFAR.

Researchers hold interviews with senior facility staff (approximately three per facility, each contributing to a single data collection form) to collect staff-reported information on facility structure, service delivery, care offered, and to ask their views about the services they offer. These staff members are also asked to provide blank service documents (including service aim, referral forms, assessment sheets and patient information sheets), where available.

#### Phase 1 data collection tool 2: Facility document collection

In order to study the level of patient-level clinical information collection and management at each facility, the existence, format and language of specific clinical documents relating to care in the facility are recorded (service aim, inwards referral criteria, incoming/outgoing referral forms, patient charging, ART protocols, care protocols, first assessment sheets, ongoing contact assessment sheets, patient records, referral follow-up forms, stock control sheet, and information to patients). Blank example documents are taken, where available, for content analysis.

#### Phase 1 data collection tool 3: Pharmacy review

Researchers record the level and place of drug stock for in-date and expired drugs separately, and if there had been previous stockouts (in-date drugs only) for various formulations of drugs commonly used in HIV care.

To complete the pharmacy review, researchers visit the pharmacy to review stocks and stock cards and complete the standard tables on current drug stocks, stockouts, stock levels and access. This is conducted with the assistance of the pharmacist (or dispenser or other staff who worked in the pharmacy).

#### Phase 1 data collection tool 4: Patient focus group discussions

Researchers lead patient discussion groups using the semi-structured interview schedule. The focus group discussions (FGD) have two main aims: to act as a validation of the senior staff interview data relating to components of care offered, and to explore aspects relating to patients' care (e.g. which components of care are valued and why, any problems in obtaining services).

FGDs are held with existing patients at each facility. Inclusion criteria are as follows: adult patients who have been under care for at least 6 weeks, who are known (by both the patient themselves and clinical staff) to be HIV positive and give informed consent to participate (following provision of an information sheet and consent form). Patients are purposively selected with the aim of obtaining a diverse group with respect to gender, age, disease stage and anti-retroviral (ARV) use.

Approximately five patients in each facility are invited to participate in the discussion group, led by the researcher. Researchers make notes on the responses to pre-specified questions on the interview schedule, and the FGD is digitally recorded as a back-up. During each FGD, demographic information is collected on participants' gender, location (urban, rural or peri-urban), age and household size. Participants also state if they have accessed specific key components of care including daily cotrimoxazole (CTX), a mosquito bednet and nutritional counselling.

### Data management and entry

Data are transferred from Phase 1 facilities immediately after collection. Quantitative data (i.e. closed questions from the senior staff interview and the pharmacy review) are double-entered by two different researchers, and validated, using EpiData v3.1. Errors in data entry and data recording are identified using consistency and logic checks, and followed-up by manual checking of questionnaires. Responses to open-ended questions and FGDs are entered into pre-formatted templates in MS Word 2003. Information from the record of documents available at the facility, and their content, are entered into tables in MS Word 2003 files.

#### Phase 1 analysis plan

### Senior staff interviews

Analysis is conducted using Stata v10 (quantitative) and NVivo v7 (open-ended questions). Frequency tables are generated for key responses, grouped by facility type where appropriate. A Spearman's rank test for correlation is conducted to test the reliability of routine data. Patient numbers are weighted to account for the stratified design used to select facilities. Thematic analysis of content is conducted on the responses to the open-ended questions [16]. The principal themes are organised into data categories and then agreed between two researchers.

### Facility document analysis

To determine the availability of the various types of service documents, a matrix is developed to record the number of facilities who report having each document, and the number and percentage of facilities that provided examples for further analysis.

Where the percentage of facilities who provided examples of documents as a proportion of those who reported such documents existed is less than 20%, or where the absolute number of documents is five or fewer, no further analysis is undertaken. Researchers conduct telephone conversations with site representatives in these cases to determine the reason for non-provision.

In those instances where the percentage of facilities who provide examples of documents as a proportion of those who reported such documents existed was equal to or greater than 20%, content analysis is undertaken. Data are extracted to common tables, and frequencies described for the number of facilities reporting each type of recording sheet, whether a sample is obtained, the specific nature of the information in the document fields reported, and described according to facility type.

### Pharmacy review

Analysis is conducted using Stata v10. Frequency tables are generated for each drug, grouped by facility type where appropriate. Data from the pharmacy review is compared with components of care offered according to the senior staff interview data in the CSRI.

### Patient focus group discussions

Information on FGD participants' background and receipt of care items is entered into a predesigned table by the researchers, transferred into an Excel spreadsheet and then merged with the Stata database using a unique identifying variable. The care received by FGD participants is integrated with the facility staff reports of care offered.

Analysis of the FGDs text is conducted using NVivo v7. In the same way as for the open-ended questions in the senior staff interviews, thematic analysis of content is conducted on the notes from the FGDs. The principal themes are independently organised into data categories and then agreed between two researchers.

#### Phase 2 Methods

### Sampling procedure for Phase 2

### Sampling health facilities

In Phase 1, the approximately 1200 facilities receiving PEPAR Care and Support funding in Kenya and Uganda were divided into three strata based on the number of patients treated in the past year, and 20 facilities selected at random from each stratum within each country. From these 60 facilities in each country, the largest six in each were selected to participate in Phase 2. The inclusion criteria for Phase 2, which applied in addition to those for Phase 1, were that facilities: recruited at least 30 new HIV patients a month; had sufficient staff with essential skills to conduct data collection; offered ongoing care and support to enable longitudinal data collection; had sufficient capacity to engage in the research.

The six largest facilities in each country were selected because they were the most likely to meet the inclusion criteria listed above, and to remain engaged in the longitudinal recruitment and data collection. Facilities were informed of the planned survey and invited to participate through the Ministries of Health in each country.

### Sampling patients

This Public Health Evaluation is not a trial, and does not aim to observe an expected effect. Therefore the Phase2 cohort study did not require statistically generated sample size calculation. The sample size of 100 patients per facility was selected based on the expected number of new patients registered per month with adequate follow-up data collection, and will be pooled at the country level.

Consecutive patients who meet the following inclusion criteria are approached for participation in the longitudinal study: 18 years of age or over; diagnosed HIV positive; patient knows of their diagnosis; has sufficient cognitive ability to answer the questions for the study; new to service or presenting with a new problem (social, psychological, spiritual or physical).

Participants give informed consent to participate following provision of an information sheet and consent form. These documents have been translated into local languages in Uganda (Kiswahili, Dholuo, Runyakitara and Luganda), and Kenya (Kiswahili, Dholuo). Information and consent form are read aloud by the health care worker for nonliterate prospective participants. Participants are reimbursed travel expenses to the facility of US$5 per research interview visit. Each of the 12 facilities recruits 100 participants.

### Data collection tools and procedure

#### Qualitative interviews

At each facility, seven patients, three family caregivers and five staff are invited to interview. The purposive sampling strategy includes a variety of staff designations with direct patient contact. Eligible participants for the patient qualitative interviews are patients of the facility who have been diagnosed as HIV positive, over 18 years of age, been under care for at least six weeks, and are not involved in the longitudinal study. These patient participants are asked for consent to approach an identified adult informal carer (i.e. family member or friend who provides assistance/support).

Patient, carer and staff participants give informed consent to participate following provision of an information sheet and consent form, which is read aloud to the interviewee by the health care worker if the interviewee is non-literate.

The semi-structured Interview schedules are designed to gain greater understanding of service use, provision and experiences from the views of the patients, their informal caregivers and the facility staff. The principal themes of enquiry within the schedules for patients and carers are experience of facility care, choice of facility, the nature and content of clinical encounters, and principal needs. Within the interviews, the full range of medical, psychological, spiritual and social domains are addressed. Initial interview transcripts will be reviewed and the topic guide revised where needed to improve clarity of questions and fully explore key issues and any emerging themes. The interview schedules, information sheets and consent forms have been translated into local languages from the English versions twice, independently, by two local researchers. Each of these versions has been translated back to English by a third researcher, with any discrepancies discussed amongst the group and an agreed translation decided.

Qualitative interviews with staff members, patients and informal carers are conducted in private (usually in consulting rooms at the care facility) in local languages as indicated above, and digitally recorded.

One facility is located in a remote part of Uganda where the most widely spoken local language is nationally very rare, and spoken by none of the study researchers. Rather than exclude an entire ethnic group from this section of the evaluation, the patient and carer interviews are conducted by a member of facility staff trained in qualitative interviewing, with the researcher present.

#### Longitudinal quantitative study

The data collection tools in the Phase 2 longitudinal study are four questionnaires, one of them (demography) used only once per participant and the others (validated tools) used four times at monthly intervals. The time points, each one month apart, are designated T0 (entry to the study), T1, T2 and T3. A 'patient pack' has been created for data collection, consisting of all the tools bound in the order they should be used, with the pages colour coded by time point, and preceded by a log page to complete the dates of interviews and a front cover with the patient's ID number. For each facility, questionnaire packs have been prepared in two languages; English and a common local language. Translation procedure for the packs is the same as for qualitative interview schedules.

Basic demographic and medical details are collected using a brief questionnaire administered at T0 (recruitment to study). Four clinical questions are asked at T1 (one month later): World Health Organization (WHO) HIV disease staging, date and result of most recent CD4 test, and date of beginning ART. These items were moved from T0 to T1 because in piloting, health staff pointed out that the information would not be available to new participants at T0, and the response rate to these questions would be higher at T1.

The APCA African POS is an adapted version of the original POS, which was developed at KCL to address the multidimensional problems of patients with incurable progressive disease and subsequently adapted around the world [17,18]. The African APCA POS was developed and validated in ten centres in six Sub-Saharan African countries in 2006 [19,20]. Its ten items address the primary physical, psychological, emotional and spiritual concerns of patients and families and employs scoring methods appropriate for a range of literacy skills. The validation study demonstrated its properties included sensitivity to change, and it has high levels of patient and clinician acceptability. The ECOG is a clinician-rated single item measure of physical performance,, also at administered at all four time points [21]. Scores range from 0 (normal activity) to 4 (unable to get out of bed). The ECOG is the most widely used performance measure [22].

The MOS-HIV is a very widely used quality of life measure and has been culturally adapted to the Ugandan HIV setting [23,24]. The 35 items address the domains of: role function; pain; physical functioning; cognitive functioning; overall health perception; mental health; vitality. The weighted subscores in these domains are then combined to produce two summary scores measuring physical health and mental health.

A version of the Client Services Receipt Inventory (CSRI) [25] has been adapted for the aims of this study and the HIV setting in Africa in order to collect information about services received by patients in the study. The CSRI records receipt of 52 components of physical, psychological, spiritual, social and preventive care, and whether they were received at the facility or from elsewhere.

With the exception of CD4 counts, data for the longitudinal quantitative study are self-reported by the participants, and recorded in the questionnaire packs provided by health care workers (HCWs) already employed at each facility.

HCWs are trained in the process of seeking informed consent and the completion of the questionnaires. A researcher maintains contact with each facility through regular visits including observing data collection, checking the use of appointment diaries and regular data entry, and delivering additional training as necessary.

Data collection (i.e. at recruitment (T0) and at three subsequent interviews a month apart) coincides with clinical appointments where possible. Once participants complete the longitudinal study, CD4 counts are extracted from patient records by the HCWs, or by the researchers themselves under the supervision of HCWs.

Piloting demonstrated that participants often could not remember the date and result of their last CD4 count. Accordingly, permission was granted by the ethics board overseeing the study to search patients' records for CD4 counts. Therefore, researchers visit each facility and transcribe CD4 counts for the study participants from their file into a specially designed form which preserves anonymity while allowing records to be linked to participants. CD4 count is the only variable data obtained in this way. The decision to refer to patient records was taken because the researchers knew the information was collected by facilities, and participants had been informed of their result, but they simply could not remember the information.

One questionnaire pack contains all the data sheets for one individual. The pages are colour-coded to indicate time points. The front cover of the pack is blank apart from the patient's name. When the final time point is complete, this page is torn out and destroyed, making the data unidentifiable. The second page includes metadata logging the progress of data collection and management for each time point.

#### Costing items

Because the provision of care is a complex area of investigation, there are potentially a number of cost components that could be accounted for. Due to the constraints of this large multi-methods study (of which economics are a component rather than the primary outcome of interest), the following key cost drivers are examined: labour (by staff type, staff salaries); medicines (ARVs, pain medicines, antibiotics, other) and their inventories (buffer stock); laboratory items (supplies and equipment); buildings (assigned market value or annual rent cost) and utilities; capital inputs (high end equipment and vehicles).

Because only the major cost drivers are included, the costs in this report are likely to be an underestimate of the real costs of providing care. Cost elements that may be significant, but which are not accounted for include: costs of developing training programmes, training of health providers, supervision, monitoring and evaluation including health information management, clinic administrative costs, drug and commodities management and maintenance and depreciation on capital assets.

As HIV care and support is provided in a clinical setting in which other non-HIV services are provided, it is necessary to estimate the proportion of some cost elements which are measured for all clinical services and attribute a share for HIV care and support. This is a common issue with the costing of services that are provided in an environment where several medical specialities are simultaneously provided separately at one facility. For labour costs, the proportion of time that staff are involved in providing care and support services is used to calculate the fraction of labour costs allocated to care and support.

Capital and building costs are allocated to HIV care and support using the proportion of all patients accounted for by HIV patients. Similarly, drug costs that are not ARVs and not for analgesia (opioids and non-opioids) are allocated to HIV care using the same proportion. Tools have been piloted and revised to maximise validity of the data collected, and for ease of data provision among facilities.

A data collection instrument was designed and tested to capture the identified cost elements in each of the Phase 2 facilities. Facility level data includes number of patients seen by staff category in a typical day, hours spent with HIV patients per week and hours worked per week by staff category; number of staff by category involved in HIV care; quantities of medications dispensed in the previous three months by drug type; numbers of laboratory tests conducted in the previous three months; information on physical buildings such as space and an equivalent in rental value of the space; utility costs per month including water, electricity, generator fuel, communications, waste disposal etc.; transport costs, fuel, costs of drivers, maintenance; clinical consumable costs per month (including gloves, syringes, cotton wool swabs, plasters, soap, sterilizing solution etc); amount spent on volunteer staff including training, travel reimbursements, payment in kind in previous three months.

Researchers gather the information required for the instrument with information provided by key informants at each facility. Key informants vary by facility but generally include the hospital administrator or manager, the accountant, clinicians, nurses and pharmacists. More than one site visit is undertaken in order to interview all relevant respondents and to complete the questionnaire.

Unit costs are required for some cost elements. These include staff (i.e. full salaries including allowances), prices of medicines and unit costs of laboratory tests. Salaries are obtained from each site and were average levels paid for each staff category. Drug costs are largely obtained from international sources such as the WHO and the *International Drug Price Indicator Guide *[26] while laboratory test costs are obtained from ATC [27].

### Data management and entry

#### Phase 2 Qualitative interviews

Interview recordings are transcribed verbatim into MS Word 2003 in the language in which they are conducted. If the interview is not conducted in English, two independent translations into English are then performed, either by the researchers or by experts from the Department of Linguistics at the local Institute of Psychology. A team of three then reconcile the two independent translations, referring back to the recorded interview if necessary, and agree upon a final version. For the three interviews conducted by a health worker, a team of three health workers at the facility translate the recordings into English, and a native-speaker linguist checks the transcript while listening to the tape. After the final translations are agreed, the recordings are destroyed.

A table containing demographic information on the participant is added to the beginning of each transcript. These data are extracted from interviews and subsequently entered into Excel tables.

#### Phase 2 Longitudinal quantitative data

Immediately after collection, data are entered into a pre-designed EpiData v3.1 database with conditional checks for internal consistency. Data entry is conducted at the health care facility by an administrative staff member trained in the use of the tools and the database. When participants have completed the final data point, the completed data collection tools are transferred to the local central research office. There, the research staff conduct a second round of all data entry and validation of both rounds of data entry. Discrepancies identified are corrected by manual checking of questionnaires, and results revalidated until the two datasets are identical. The CD4 information from patient records is entered into a separate EpiData database and merged into the main dataset.

#### Phase 2 Costing items

The data are entered into predesigned Excel spreadsheets. Analysis, including creation of graphs and linear regression, is conducted in Excel workbooks with data drawn together from the different sheets.

#### Phase 2 Analysis plan

The three main sources of data (longitudinal quantitative study, qualitative interviews and costing) are analysed separately. Analysis plans for each data component are outlined below.

### Qualitative interviews

The interview verbatim transcripts are imported from Word into NVivo 7 for coding and analysis. Information on interviewees' age, gender, household location, family size, profession (for staff), whether they were receiving ART (for patients) and relationship to patient (for carers) are extracted into an Excel table, subsequently imported into NVivo. Identifying information such as names of individuals or care facilities are removed from transcripts. Thematic analysis of content is conducted concurrently on the patient, carer and staff interviews to enable multiple perspectives on each coded theme.

Three coding frames are developed and subsequently combined into a single version applied to the transcripts for the remainder of the coding. One coding frame is developed in Uganda by the team of local researchers who had conducted the interviews, and a second by the Kenyan researchers. The third coding frame is developed at KCL in London. The intention is to explore cross-cultural differences and similarities in coding, and to generate a definitive coding frame that reflects local understanding of the data and scientific rigour.

In each three-person country research team, every researcher codes nine randomly selected interviews (three with a patient, three with a member of staff and two with a carer), creating hierarchical codes. The local team members agree on a coding frame by discussion, comparison and consensus. The three teams then meet and a unified coding frame is developed, combining the strengths of each country-level frame. Each code is reviewed for internal consistency and given an agreed definition to ensure it is applied using a standard meaning by each researcher. The researchers are trained in the use of NVivo 7 and in application of the new coding frame, which is applied to the entire dataset.

### Quantitative longitudinal patient data

The primary outcomes are the physical and mental health summary scores derived from the MOS-HIV. Secondary outcomes are individual POS item scores, and a combined total POS score. Individual POS item scores are not expected to be parametric and are summarised using the median and inter-quartile range. A a p-value of 0.05 will be used to determine significance in all statistical tests except for the decision to include variables in multivariate analysis, when 0.1 will be used. Initial cross-sectional descriptive analysis of the data will be conducted first.

Time points will be compressed and recoded if necessary to make the most efficient use of the data. For example, if a participant is recorded to have completed T0, T2 and T3 but missed T1, then T1 is deleted from the record and T2 and T3 renumbered T1 and T2, to obtain a continuous series of three points.

Clinical variables (CD4 count, HIV disease stage, physical function) and demographic characteristics (age, gender, location of home, education, number of dependants) of participants will be described for the total sample and by country and facility. Variables relating to socioeconomic status will be incorporated into a principal components analysis to generate a single factor, and this will be split into quintiles of relative wealth[28].

For univariate analysis of demographic/clinical characteristics with primary outcomes at baseline, ANOVA will be used for ordinal variables and linear regression for continuous.

The 52 components of care recorded in the CSRI will be grouped into care themes according to the issues they address and the way in which they are delivered. Care themes are listed following.

Spiritual: visit by a religious leader, prayer with patients, and contact with traditional healer. Spiritual care is a distinct aspect of palliative care. Staff praying with patients, and a visit from a religious leader, are the most common types of spiritual care provided through health facilities. Many people with HIV visit traditional healers[29] and the care delivered by them fits the PEPFAR definition of spiritual care being sensitive to individual and community culture [30].

Counselling and advice: pre-and post-test counselling, adherence counselling, family planning counselling, patient HIV support groups, family counselling and psychiatric therapy. This theme comprises all 'talking therapies'. It is sometimes difficult to distinguish counselling as listening and responding to the patient's worries and concerns from counselling as didactic imparting of information. VCT, for example, is a strategy of both prevention and care, assessed for its efficacy in reducing risk behaviour and HIV transmission[31].

Nursing: wound care and other nursing care.

Pain management: assessment of pain and provision of strong and weak opioids or non-opioid analgesics and treatment for neuropathic pain.

Pain is a common symptom in HIV [32] and all five components in this group are necessary for its relief. The WHO pain ladder [33] outlines the need for non-opioid and opioid analgesics until pain has been controlled. Neuropathic pain, which is particularly common in HIV [34] is caused by damage to nerves and does not respond to traditional pain medication.

Symptom management: treatment for anxiety/depression, nausea/vomiting, skin rash/itching, diarrhoea, laxatives, thrush, oral candidiasis, cryptococcus, other fungal infections, herpes, malaria and other opportunistic infections. The components in this theme were usually defined by the symptom treated, rather than the underlying cause or pathogen, because the cause of a symptom is often not known in HIV disease [35]. All these physical symptoms and conditions are common in HIV [35,36].

Nutrition: food, multivitamins, nutritional advice, safe drinking water, therapeutic feeding for malnutrition. Poor nutrition comprises two problems: lack of macronutrients (wasting, malnutrition) and lack of micronutrients (vitamins and minerals). Both of these predispose individuals with HIV to infections and ill health. Lack of food is the most fundamental level of poverty.

Social: employment training/income generating activities (IGA), home help, household items, legal services, memory book work, loans/microfinance. The social group components were selected after advice from USG country mission teams. Memory book work was allocated to the social care group because it aims to reduce internalised stigma and improve relations between family members.

Prevention: prevention with positives, condoms, ITNs, infection control training, isoniazid for TB prophylaxis. This care group includes both components to protect the person with HIV from other infections, and components to prevent them from infecting others with HIV. Prevention with positives is the general name for a package of care designed to encourage behaviour change (condom use, reduction of partners, and revealing HIV status). Condoms prevent further infection and also protect the individual from other strains of HIV and from other STIs such as herpes. Insecticide-treated nets protect against malaria, which is more common and more aggressive in people with HIV[37], and the TB drug isoniazid can be used as a prophylactic for those at high risk of TB.

ART: ARVs and assessment of ARV treatment. Antiretroviral therapy includes regular assessment to observe signs of developing resistance, toxicity and side effects.

CTX: daily CTX. PEPFAR guidelines encourage daily CTX prophylaxis for everyone with HIV. It is a broad-spectrum antibiotic proven to reduce morbidity and mortality in people with HIV[38,39]. At each interview participants are asked whether they had taken CTX on the previous day and whether they had been given daily prophylactic CTX in the last month. These answers are compared to test adherence.

TB: TB treatment. TB treatment is listed separately from treatment for other symptoms and infection, for two reasons. Firstly, it is the leading cause of death for people with HIV in Africa[40]. Secondly, the course of treatment lasts for four to six months, long after symptoms have resolved. The full course of treatment must be completed to prevent resistance and recurrence.

Baseline outcomes, demographic variables and care theme delivery will be compared between the facilities.

To test the hypothesis that participants who receive antiretroviral therapy or TB treatment at T1 (a month after recruitment) are likely to have more advanced disease, physical and mental health, and CD4 count, will be compared for recipients and non-recipients using t-tests. Physical and mental health outcomes will also be compared for those who attend with and without a carer, to test the hypothesis that carers are more likely to accompany those in poorer health.

The longitudinal outcome analysis will be conducted as follows. Mean and 95% confidence intervals for physical and mental health score are plotted over time as a graphical representation of change in health states.

Longitudinal multilevel modelling is an approach which makes the most efficient use of data collected over time. Unlike most statistical tests, it includes all time points at once, which both reduces the number of tests to be carried out (making false positive results less likely) and allows change to be modelled as a continuous effect. This means that rather than simply finding variables which are associated with any change in outcome, the magnitude of the change can also be considered.

A common problem in longitudinal studies of health outcomes is that patients with the worst health are the most likely to leave the study and be lost to follow-up, so that a comparison between the beginning and end of the study could find improved outcomes only because a proportion of those with poor health would not contribute to the later timepoint. Longitudinal analysis does not have this bias because all participants can be included whether they complete the study or not. Additionally, longitudinal analysis can reveal patterns over time which would not be identified using traditional methods.

The technique adopted in this study was multilevel mixed-effects linear regression, selected because it allows data to be clustered at two levels, by individual and by facility [41]. Many longitudinal studies suffer from the bias caused by the most unwell individuals being most likely to leave the study. To determine whether this bias was present, t-tests are used to compare the mean physical and mental health scores of those who completed all four observations with those who missed at least one. Traditional analysis of longitudinal data involves comparing the earliest observation with the last, so any difference in the scores of completers versus non-completers would bias the findings.

In addition, the same tests are used to compare the mean scores of those who only completed a single observation with those who completed two or more. This is to test the suitability of multi-level modelling, which is explained below. Multi-level modelling uses all data points except the first one, so anyone who only completed one observation would be excluded and it is necessary to test whether this would also cause a bias. For the same reason, mean change over time is reported as the mean of all individual score changes, rather than mean health score at one time point subtracted from mean health score at another.

Multilevel modelling is carried out using the Stata *xtmixed *command function. Outcomes are physical health score and mental health score, measured up to three times at monthly intervals. Baseline score is incorporated as a covariate and not as an outcome. Models included levels for facility and individual. The only random effect is time point (interview number, rather than actual time interval), which is allowed to have a random coefficient at the individual level. Other covariates are fixed. Demographic and care theme covariates are constant over time, receipt of ART and TB treatment varied. The default independent covariance structure is used. Variance and standard error of variance are reported for random-effects parameter estimates.

This analysis is repeated with the 20% of participants who had the lowest physical health score at T0, and with the 20% who had the lowest mental health score at T0, to determine whether the effect of improved outcomes over time extended to those in greatest need. For the APCA African POS, score distribution at each timepoint is tabulated for those who scored 0 (worst possible problem) on the items relating to pain and symptoms. This simple approach is adopted because a very few people scoring 0 on these items could introduce bias. The items for pain and symptoms are selected because people with complex, intractable problems in advanced disease may not experience improvement although average scores for the population increase.

The multilevel models will be repeated several times, each time with the addition of a single demographic covariate. Models will also be developed with ART receipt and TB treatment, which will vary over time.

Examining the relationship between health outcomes and care received is complicated by the potential bias that those in the worst health would probably receive the most care, whereas a lack of care could mean either no need of it, or lack of appropriate provision. To avoid this problem, a variable representing available care is required, which might have a closer association with health outcomes than the level of care individually received. Availability of care is defined as the percentage of individuals at a facility who receive care in a particular theme. Care themes are used rather than individual components in order to reduce the number of variables needed in the model and ensure stability. For example, the variable 'psychological care', contains information on the percentage of people, per facility, who receive at least one component of psychological care at T1, T2 or T3. T0 is excluded because the model analyses change from T0 onwards.

Each of the care themes is included one by one in a univariate multilevel model as described above; with the difference that care theme has only twelve values, one for each facility, and does not change over time.

Following this, all individual-level and facility-level covariates associated with outcome at the 10% level in univariate analysis are taken forward into a multivariate model and eliminated in a downward stepwise procedure if the association is lost. It is usual to use 10% as the acceptance level with stepwise downward regression to avoid dismissing variables too early.

### Costing study

The purpose of the costing survey is to develop understanding of the factors which influence per-patient costs, and to guide resource allocation, for example by observing economies of scale. Analysis is conducted using an Excel spreadsheet. Most data are collected in local currency (Kenyan and Ugandan shillings), and converted to US dollars at the current exchange rates. Since only twelve facilities are included and these facilities were not meant to be representative of all sites, all results are reported per facility without aggregation across the sample. The average costs per patient for one year of care and support are calculated using aggregated average costs per patient for each of the main components of care for a year.

### Data integration

The final step of analysis is the integration of The Phase 1 and Phase 2 data are integrated to enable the outcome and cost data to be appraised in the light of the facility configuration data

## Discussion

This substantive study will provide novel data to inform the provision of HIV care and support programmes in Sub-Saharan Africa. The dual phases and mixed methods allow triangulation and integration of diverse types and sources of data to better determine patient outcomes and factors associated with patient and family experience of HIV care.

We believe that there are several elements to the design and implementation of this protocol that are original and offer methodological advantages. Firstly, the aims objectives and associated data collection tools have been informed by a large collaboration between practitioners, policy and programme designers, implementing partners, academics and Ministry of Health representatives across 3 continents. This has greatly improved the potential data demand and utilisation of the study. Second, all research staff involved in data collection and working with facilities are indigenous African researchers, and considerable capacity has been built through ongoing training and the conduct of this large study. Third, the novel mixed methods approach brings together an integrated mixture of outcome measurement, longitudinal evaluation, qualitative approaches, health economics, document analysis and drug availability review to evaluate HIV care and support. Fourth, the careful attention to multiprofessional inclusivity and consultation, and piloting of tools, has maximised the feasibility of this study where many logistical challenges are presented [42]. We have encountered ethnic and political violence and unrest, lack of available transport to some regions, and temporary closure of services to patients, although these challenges have been overcome. Fifth, we have paid considerable attention to data demand and utilisation, which has encouraged us to initiate processes of consultation and feedback at each stage of protocol design and to identify the data needs of our stakeholders. Sixth, we have been clear that this evaluation does not aim to compare outcomes between facilities or countries (e.g. to identify facilities performing less well), as it is important to encourage participation in this type of outcome evaluation without introducing objectives that may be interpreted as critical or competitive. We have generated a regular study newsletter to promote communication and information flow between research sites and partners. Seventh, we have also taken steps to reduce potential bias in the methods, for example by forward and back translation of tools and selection of person-centred outcome tools that have been locally validated. Our analytical procedures have attempted to identify bias commonly identified in this type of data for example by potential attrition of the sickest, and the use of longitudinal approaches that maximise use of the data. Error has been reduced through double entry of all quantitative data and careful translation of qualitative data. Lastly, we offer reimbursement to participants of $5 for each research interview, which is felt to not be large enough to be seen as an unethical inducement to participate in the study, but is enough to comfortably cover costs of attending additional research interviews. The use of payments to take part in longitudinal research in low income countries is advocated, not least due to emerging evidence that a key reason for non-adherence to ART is inability to afford to attend HIV clinics [43-45].

Analysis is currently underway. We believe that the presentation of this protocol offers greater transparency in the aims, objectives and analysis of our study, and will permit greater focus on the reporting of our large dataset.

## Competing interests

The authors declare that they have no competing interests.

## Authors' contributions

The Principal Investigators are RH and IJH. All authors contributed to the design and protocol development. All authors have read and approved the final manuscript.

## Pre-publication history

The pre-publication history for this paper can be accessed here:

http://www.biomedcentral.com/1471-2458/10/584/prepub
